# Depressive disorders in the elderly and dementia: An update

**DOI:** 10.1590/1980-57642020dn14-010001

**Published:** 2020

**Authors:** Natália S. Dias, Izabela G. Barbosa, Weihong Kuang, Antonio L. Teixeira

**Affiliations:** 1 Neuroscience Program, Institute of Biological Sciences, Universidade Federal de Minas Gerais, Belo Horizonte, MG, Brazil.; 2 Department of Mental Health, School of Medicine, Universidade Federal de Minas Gerais, Belo Horizonte, MG, Brazil.; 3 Department of Psychiatry, West China Hospital of Sichuan University, Chengdu, China.; 4 Department of Psychiatry and Behavioral Sciences, UT Health, Houston, United States.; 5 Instituto de Ensino e Pesquisa, Santa Casa BH, Belo Horizonte, MG, Brazil.

**Keywords:** depression, dementia, Alzheimer’s disease, differential diagnosis, therapeutics, depressão, demência, Alzheimer, diagnóstico diferencial, terapêutica

## Abstract

The relationship between depressive disorders in the elderly and dementia, particularly Alzheimer’s disease (AD), is highly complex. While the nature of this relationship is still a matter of debate, differential diagnosis and treatment remain a great clinical challenge. We review recent findings on the conundrum of depressive disorders in the elderly and AD. There is a biological continuum between depressive disorders in the elderly – or at least a subgroup of them – and AD. While elderly subjects with depression and patients with AD exhibit higher circulating levels of pro-inflammatory molecules and lower BDNF than matched controls, CSF levels of Aβ42 can discriminate AD from depressive disorders in the elderly. The role of antidepressant treatment as a strategy to minimize the risk of AD remains to be established.

## RESUMO

A relação entre transtornos depressivos em idosos e demência, particularmente a doença de Alzheimer (DA), é altamente complexa. Embora a natureza desse relacionamento ainda seja motivo de debate, o diagnóstico e o tratamento diferenciais continuam sendo um grande desafio clínico. Revisamos descobertas recentes sobre o dilema de transtornos depressivos em idosos e DA. Existe um contínuo biológico entre os transtornos depressivos em idosos – ou pelo menos um subgrupo deles – e a DA. Enquanto indivíduos idosos com depressão e pacientes com DA exibem níveis circulantes mais altos de moléculas pró-inflamatórias e menor BDNF do que os controles correspondentes, os níveis de Aβ42 no LCR podem discriminar a DA de distúrbios depressivos em idosos. O papel do tratamento antidepressivo como estratégia para minimizar o risco de DA ainda precisa ser estabelecido.

The world’s population is ageing rapidly. The proportion of the world’s older adults is estimated to almost double between 2015 and 2050.^1^ In absolute terms, this is an expected increase from 900 million to 2 billion people over the age of 60.^1^

The prevalence of depressive disorders among older adults is around 35%.^2^ Actually, depressive disorders – both major depressive disorder (MDD) and subthreshold depressive symptoms – are the most common psychiatric disorders in the elderly.^3^ Moreover, a Brazilian study showed that the frequency of depressive symptoms in people between 60 to 79 years of age is 30% higher than with young adults.^4^ Depressive disorders also have a significant social impact, and specifically MDD accounts for 5.7% of years lived with disability (YLDs) among people older than 60 years.^1^

Depressive disorders in the elderly can be classified according to the age of emergence of the first mood episode.^5^ Early-onset depression (EoD) is defined by the first depressive disorder starting before 60 years of age, while late-onset depression (LoD) refers to the first depressive disorder onset after 60 years of age.^5^

In the elderly, depressive disorders, particularly LoD, are marked by apathy, psychomotor changes and cognitve impairment.^6^ Differentiating cognitive deficits secondary to depressive disorders from dementia with depressive symptoms remains a great clinical challenge. This is complicated by the fact that depressive disorders in the elderly have been defined as risk factor or prodrome of neurodegenerative diseases, possibly reflecting underlying vascular and/or degenerative processes.^7^

This review aims to discuss clinical presentation, pathophysiological mechanisms and treatment of depressive disordes in the elderly. For this narrative review, we searched the pertinent literature on the Pubmed database until November 2019, focusing on meta-analyses, systematic reviews and studies published in English in the last five years. We also included historically relevant studies.

## DEPRESSIVE DISORDERS IN THE ELDERLY AND COGNITIVE DEFICIT: THE CLINICAL CHALLENGES

Cognitive impairment is a definig feature of depressive disorders according to the DSM-5^8^ and ICD11.^9^ However, when elderly patients with depressive disorders exhibit marked cognitive dysfunction and even functional decline, they might be diagnosed as “pseudodementia”. This is an old and highly disputable concept that underscores the cognitive symptoms in the context of mood disorders.

Cognitive deficits related to depressive disorders are classically marked by executive dysfunction, including problem resolution and planning, flexibility, decision making ability and inibitory control,^10^^,^ 11 and impairment in selective and sustained attention, semantic and fonemic fluency.^11^ In the elderly, EoD has been associated with episodic memory deficits and bilateral hippocampus atrophy.^12^^,^ 13 This finding goes in line with the epidemiological evidence implicating depression as a risk factor for Alzheimer’s disease.^14^ Conversely, LoD has been associated with more executive disfunction and prominent cerebrovascular lesions in pre-frontal regions, *i.e.* white matter hyperintensities in magnetic ressonance imaging.^12^^,^ 15 ^,^ 16 More recent studies have shown more severe memory impairment and hippocampal volume loss in LoD compared to EoD,^11^^,^ 17 suggesting that not only vascular burden but also neurodegenerative changes might be implicated in the pathogenesis of LoD.

Depressive disorders have been associated with cognitive impairment even when euthymic state is achieved.^18^ A recent study showed that remitted MDD patients had a cognitive performance between healthy controls and non-remitted MDD patients.^19^ In the elderly, depressive disorders have also been associated with cognitive deficits after mood symptom remission.^20^^,^ 21 Interestingly, cognitive impairment seems to be mediated by apathy in elderly subjects with MDD as in neurodegenerative diseases like AD and Parkinson’s disease.^21^

There is considerable overlap in clinical presentation, neuropsychological performance and even neuroimaging findings between elderly patients with depressive disorders and patients with dementia.^7^ The differential diagnosis of these two conditions can be challenging, requiring careful clinical follow-up with neuropsychological assessment and, if availabe, CSF and PET neuroimaging tests. Understanding the biological interaction between depression and dementia and identifing putative biomarkers could improve both the diagnosis process and the treatment of depressive disorders in the elderly.

## DEPRESSIVE DISORDERS: RISK FACTOR OR A PRODROME OF DEMENTIA

In the past two decades, several populational studies have shown that depressive disorders are risk factor for cognitive decline and dementia, especially AD.^22^^,^ 23 A recent metanalysis including longitudinal studies with 49,612 subjects further promoted the association between depressive disorders and dementia with 2.53 fold-increased risk for vascular dementia and 1.85 fold-increased risk for AD.^24^ It is worth mentioning that most studies in this meta-analysis did not clearly differentiate between EoD and LoD.

Otherwise, two recent community-based studies suggested that depressive symptoms are prodrome of dementia. Mirza and colaborators (2015)^25^ performed a 11 year cohort study evaluating depressive symptoms in 3,325 non-demented participants. Five trajectories of depressive symptoms were identified: stable low depression scores (2441 [73%]); moderately high starting scores but then remitting (369 [11%]); low starting scores, increasing, then remitting (170 [5%]); low starting scores that steadily increased (255 [8%]); and persistent high scores (90 [3%]). Increased risk of dementia was observed in patients with steadily increasing depressive symptoms (8%), but not in the other patterns of depressive symptoms.^25^ These findings are consistent with the hypothesis that a specific pattern of depression presentation later in life might be seen as a prodrome of dementia. Indeed, depressive symptoms might lie in a continuum between subclinical cognitive impairment and overt dementia. In a 28 year follow-up cohort with 10,308 participants, Singh-Manoux and colaborators (2017)^26^ also showed that depressive symptoms can be seen as a prodrome of dementia and initiate ten years before the diagnosis of dementia.^26^

Taking into account that the great majority of neurodegenerative diseases develops throughout many years, it is conceivable that depressive symptoms might preceed cognitive decline and other clinical features.^27^^,^ 28 This supports the idea that depressive disorders in elderly population, especially LoD, is a prodrome than a risk factor for dementia. It is worth noticing that psychiatric symptoms, like depression, anxiety, euphoria and irritability can occur in patients with the established diagnosis dementia.^29^

## PUTATIVE MECHANISMS

Elderly patients with depressive disorder exhibit increased cortisol levels in comparison with age-matched controls,^30^ suggesting an impairment in the hypothalamic-pituitary-adrenal (HPA) axis.^30^ Cortisol differences during middle and older adulthood is not significantly different, but among individuals with depression, cortisol levels seemed to increase with age.^31^ Several studies have shown that increased cortisol levels are associated with hippocampus atrophy and cognitive impairment.^32^^-^ 34 Thus, the HPA axis dysfunction might contribute to the amnestic deficits shown in elderly patients with depressive disorders.^30^

Several studies have also reported association between inflammatory markers imbalance and depressive disorders. Patients with depressive disorders present increased circulating levels of proinflammatory molecules, such as interleukin (IL) – 6, tumoral necrosis factor (TNF), monocyte chemoattractant protein-1 (MCP-1) and C-reactive protein (CRP).^35^^,^ 36 The ageing process itself is associated with innate and adaptative immunity changes, collectively called immunosenescence, including low grade chronic inflammation and immune response polarization towards a Th2 profile.^30^ The presence of depression in the elderly would add further burden to the ageing process, being associated with even highter levels of pro-inflammatory molecules. A systematic review found that high IL-8, IL-6 and TNF levels might be potential biomarkers for depressive disorders in the elderly.^37^ A recent exploratory cross-sectional study showed that increased vascular endothelial growth factor (VEGF), IL-7, MCP-1, TNF and IL-1β plasma levels predicted with 95.1% of accuracy the diagnosis of depressive disorder in elderly subjects.^38^ One study evaluating 119 patients with LoD and 231 patients with EoD showed increased CRP plasma levels in LoD, and this was related with higher social stressors among LoD cases.^39^

Dementia, particularly AD, has been associated with increased peripheral proinflammatory profile as well. Patients with AD present increased IL-1β, IL-2, IL-6, IL-18, soluble TNF-receptor (sTNF-R) 1 and 2, high sensitivity CPR (hsCPR), interferon (IFN) – γ, C-X-C motif chemokine (CXCL) – 10, epidermal growth factor (EGF), vascular cell adhesion molecule (VCAM) – 1, α1-antichymotrypsin and transferrin compared to age-matched controls.^40^ These mediators might play a direct role on cognition. Moreover, IL-6 blood levels have been inversely correlated with the Mini-Mental State Examination (MMSE) scores in patients with dementia.^40^ There is no study directly comparing the immune profile in depressive disorders in elderly versus dementia.

The brain-derived neurotrophic factor (BDNF), a neurotrophin expressed in the hippocampus, plays an important role in neuronal survival, sinaptic integrity, and neuroplasticity.^41^ Decreased BDNF levels have been systematically associated with cognitve impairment^42^^,^ 43 and dementia.^44^^-^ 46 Elderly patients with depressive disorders also demonstrated decreased BDNF levels.^47^^,^ 48

Patients with AD have low β-amyloid level protein and high total tau and phosphorilated tau in the cerebrospinal fluid (CSF).^49^ The CSF levels of the isoform with 42 aminoacids of β-amyloid protein and tau protein (total tau and phosphorilated tau) are strongly related with AD neuropathology and are considered the gold-standard biomarkers in differentiating AD from dementia of other causes and psychiatric disorders,^49^^,^ 50 as depicted in Table 1.

**Table 1:** Cerebrospinal fluid biomarkers in depressive disorders in the elderly, neurodegenerative disorders and psychiatric disorders.

	β-amyloid	p-tau	total tau
**AD**	↓↓	↑↑	↑↑
**FTD**	↓	=	↑
**LBD**	↓	↑	↑
**VD**	↓	=	=
**MDD**	↓	=	=

p-tau: phosphorilated tau; AD: Alzheimer's Disease; LBD: Lewy's body dementia; FTD: frontotemporal dementia; VD: vascular dementia; MDD: major depressive disorder (in late-life). Adapted from Schoonenboom et al., 2011; with information from Nascimento et al., 2015 and Brown et al., 2016.

The current knowledge on the dynamics of Aβ peptides in the CSF and plasma of depressive disorders in the elderly is limited. A metanalysis including 142 elderly patients with depressive disorders showed decreased Aβ42 CSF levels when compared with elderly controls.^49^ However, Aβ42 CSF levels are higher^51^^-^ 53 in older adults with depressive disorders in comparison with patients with AD. Total tau and phosphorilated tau levels did not differ between elderly patients with depressive disorders and controls in serum and/or plasma^55^ and total tau and phosphorilated tau levels in the CSF are decreased in elderly with depressive disorders in comparison with patients with AD^51^^-^ 57. It is worth mentioning that these metanalytical studies did not undertake subgroup analysis taking into account EoD and LoD. This type of analysis would provide relevant information on the link between AD and a specific subgroup of late life depressive disorder.

Changes in CSF biomarkers in depressed elderly patients are in the same direction of changes observed in AD patients. These findings might sugget a shared neurobiology between depressive disorders in the elderly population and AD. Another hypothesis is that AD in the early stage and depressive disorders in the elderly might be in the same continuum.

Cerebrovascular disease is another mechanism potentially associated with depressive disorders in the elderly. It has been classicaly associated with a subtype of depressive disorder characterized by a distinct clinical presentation: psychomotor slowing, lack of iniciative and apathy, absence of a family history of depression, a medical history of hypertension and cognitive impairment, and typical neuroimaging findings.^58^ Though not formally recorded in psychiatric diagnostic manuals, “vascular depression” has been a well accepted construct in the research setting.^58^

## TREATMENT

Psychotheraphy associated with antidepressant are the treatment of choice for depressive disorders in the elderly.^59^

Whether antidepressants improve cognitive performance in the elderly is a matter of debate. A recent metanalysis of nine placebo-controlled randomized trials including a total of 2,550 participants demonstrated that antidepressant treatment was associated with increased psychomotor speed and better performance on delayed recall test.^60^ On the other hand, a community-based cohort study including 7,381 participants aged 65 years and over showed that patients taking tricyclic antidepressants (TCA) had impairment at verbal fluency, visual memory and psychomotor speed tests.^57^ Conversely, selective serotonin reuptake inhibitors (SSRI) were associated with impairment at verbal fluency and psychomotor speed tests. Besides highlighting the potential negative effects on cognition of antidepressants, this study showed that different classes of antidepressants might influence different cognitive parameters.^61^

**Figure 1:**
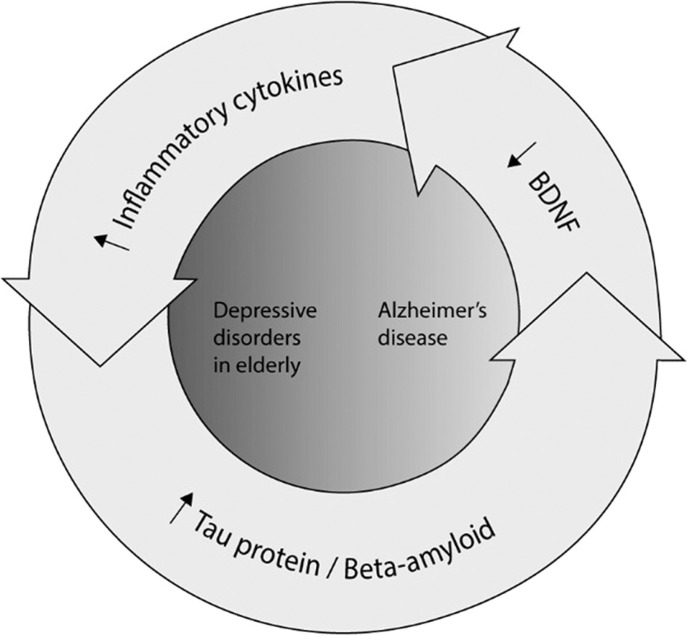
The complex interplay of biomarkers in the depressive disorders – Alzheimer’s disease continuum.

A recent review suggests that antidepressants may attenuate AD risk and, particularly, SSRIs may be effective in delaying AD onset.^62^ Unfortunately, studies reviewed are limited by their observational nature, lack of randomization and confounding biases,^62^ like the prescription of antidepressants for elderly depressed patients – that could be already in a prodromal stage of AD.

The prescription of anticholinesterase agents as add on drugs for depressive disorder in elderly patients is controversial. Donepezil as add on drug to depressive disorder in elderly patients does not seem to influence cognitive performance and the dementia conversion rate.^63^^,^ 64

A last important question must be adressed. Is there any evidence that the prescription of antidepressants might help to treat depressive disorders in patients with dementia? While the prescription of antidepressants in patients with dementia is widespread, recent metanalyses have shown that antidepressants present little or no effect on depressive symptoms, cognitive functioning and activities of daily living in this specific population.^65^ Therefore, a more critical approach is need and clinicians must consider not only this lack of evidence but also that antidepressants might cause adverse events and interact with other medications.^65^

## CONCLUSIONS

Older age is a consistent and important risk factor for a poorer course of depressive disorders.^66^ Depressive disorders in the elderly and AD present common clinical symptoms that might dim the diagnosis of both conditions. Moreover, biomarkers suggest shared neurobiological mechanisms between AD and depressive disorders. The variable terminology and classificatory systems,^67^ alongside methodological heterogeneity are still important obstacles to the field advance. More studies comparing blood-based and CSF markers, neuroimaging and neuropsychological performances in elderly patients with depressive disorders and patients with AD are needed. The identification and validation of biomarkers would help in the diagnostic process and possibly in the elegibility process for therapeutic interventions.^68^
